# Evaluation of morphological and psychological outcomes after sub-brow blepharoplasty

**DOI:** 10.3389/fsurg.2025.1658806

**Published:** 2025-09-23

**Authors:** Tengfei Wang, Yunke Li, Mengru Pang

**Affiliations:** ^1^ Department of Burns and Plastic Surgery, The Affiliated Hospital of Guizhou Medical University, Guiyang, China; ^2^ Department of Plastic and Burn Surgery, Zhuhai People’s Hospital, Zhuhai, China

**Keywords:** sub-brow blepharoplasty, eyebrow height, eyebrow-eye integration, facial aesthetics, social appearance anxiety

## Abstract

**Purpose:**

Periorbital aging commonly presents with upper eyelid laxity and brow descent. Sub-brow blepharoplasty is a promising surgical intervention to address these concerns. This study aimed to evaluate both morphological and psychological outcomes of sub-brow blepharoplasty in a Chinese population.

**Methods:**

Sixty-four patients undergoing sub-brow blepharoplasty at the Plastic Surgery Department of Guizhou Medical University (January 2022–October 2023) were enrolled. All surgeries were performed by the same surgeon. Standardized follow-ups occurred postoperatively at T1 (immediately), T2 (1 month), and T3 (6 months), with preoperative evaluation at T0. Morphological changes were assessed through standardized photography, measuring brow, eyelid, and canthal positions. Psychological outcomes were assessed using the Social Appearance Anxiety Scale (SAAS). Patient satisfaction was recorded at final follow-up.

**Results:**

Of the 64 patients, 49 (98 eyes) completed all follow-ups (mean follow-up: 7.5 months; follow-up rate: 76.56%). Significant improvements were observed in all morphological indices and SAAS scores postoperatively (*P* < 0.05). No postoperative complications occurred. At final follow-up, 71.43% of patients were extremely satisfied, 22.45% satisfied, and 6.12% neutral—yielding a 93.88% overall satisfaction rate.

**Conclusion:**

Sub-brow blepharoplasty significantly improves eyebrow position, facial aesthetics, and reduces social appearance anxiety with high patient satisfaction and minimal risk.

## Introduction

Aging is an inevitable natural process, and as individuals' facial age progresses, aesthetic changes in the periorbital area include eyebrow ptosis, lateral temporal-orbital ptosis accompanied by ptosis of the upper eyelid, and other related alterations (1, 2). The muscles controlling eyebrow movement express a wide range of emotions and gestures that are easily recognizable. Well-aligned eyebrows, paired with a relaxed, smiling mouth indicate an alert and rested state. A lateral decline of the eyebrows conveys sadness, while a medial incline suggests anger. Raised eyebrows signal surprise, whereas low-positioned eyebrows reflect exhaustion (3). The signs of aging in the eye and facial areas often emerge before full maturity, prompting many people to seek early aesthetic interventions (4, 5). Evidence suggests significant differences in the patterns of periorbital aging among different genders and ethnic groups. The Chinese Han women typically experience accelerated aging of the eyelid after 50 years of age (6, 7). Eyebrows, with their dual functional (communicative) and aesthetic attributes in the complex structure of the human face, significantly impact an individual's appearance. Eyebrow ptosis is a common condition, which occurs more frequently in elderly populations and smokers and disrupts the facial appearance of individuals. Although sometimes used interchangeably, “eyebrow ptosis correction” involves true brow elevation, whereas sub-brow blepharoplasty targets upper eyelid laxity by resecting skin below the brow without elevating the eyebrow itself (8). In today's society with high aesthetic standards, correction, and treatment are often required (3).

Currently, surgical interventions are commonly employed to address eyebrow ptosis, with brow lift techniques specifically designed to elevate the brow. In contrast, sub-brow blepharoplasty primarily targets upper eyelid redundancy and may not significantly elevate the brow position. Nonetheless, sub-brow blepharoplasty is a reliable and reproducible method for improving upper eyelid aesthetics in selected patients (9). However, the optimal choice of surgical techniques in clinical practice remains a topic of debate. Although the demand for aesthetic procedures is rising, research on the specific impact of sub-brow blepharoplasty on ocular and facial aesthetic morphology remains limited. To address this gap, this study prospectively analyzed 64 patients who underwent sub-brow blepharoplasty in the Plastic Surgery Department of Hospital of Guizhou Medical University between January 2022 and October 2023.

## Methodology

### Ethical approval

This study was approved by the Ethics Committee of The Affiliated Hospital of Guizhou Medical University [Approval No. (2020) 281]. All participants signed written informed consent forms.

### Study design

This study was a prospective analysis of 64 patients with upper eyelid skin laxity treated at the Plastic Surgery Department of Hospital of Guizhou Medical University between January 2022 and October 2023. All patients were female, aged 32–60 years, and 24 patients had undesirable eyebrow tattoos. The same surgeon performed all surgeries. The study strictly adhered to the Declaration of Helsinki and was approved by the hospital's ethics committee. Inclusion criteria included (1) patients with varying degrees of upper eyelid laxity and surgical indications; (2) patients with normal communication abilities. Exclusion criteria included (1) patients with a history of previous facial surgery; (2) patients who had previously received botulinum toxin injections; (3) patients with severe underlying medical conditions that precluded tolerance of the correction procedure; (4) patients with myasthenia gravis, congenital ptosis of the upper eyelid, or abnormal eyebrow height (i.e., significantly higher or lower than the average population norm).

### Surgical technique

All patients underwent sub-brow blepharoplasty, designed to address upper eyelid skin laxity rather than true eyebrow elevation. The design of the incision line was based on the patient's original eyebrow shape and personal preferences, ensuring the preservation of the brow head, the upper half of the brow body, and the brow peak, however, the brow tail was adjusted according to the target eyebrow shape for retention or excision. For patients with pre-existing or undesirable eyebrow tattoos, incision lines were strategically planned to exclude visibly tattooed areas, thereby improving the final aesthetic outcome. For patients with lateral brow sagging, more tissue was removed as needed. The incision started at the brow head, ran obliquely along the lower edge, peaked at 0.5–1 cm, and extended outward without crossing the nasal ala-lateral canthus line. The lower line formed a semi-arc, creating a 1.0–1.5 cm fusiform excision to prevent traction, wrinkles, and ectropion while ensuring a smooth double eyelid line.

The patient was placed supine, disinfected, draped, and given local anesthesia with 0.5% lidocaine. A vertical incision was made along the fusiform line. Superficial orbicularis oculi muscle and redundant skin/subcutaneous tissue were sharply excised to tighten the upper eyelid fold. In patients with clinically significant lateral brow sagging (brow ptosis), a small 1 cm incision at the lateral brow tail allowed mobilization of the sub-brow fat pad. This pad was attached to the supraorbital periosteum with a 4-0 absorbable suture to prevent postoperative descent. In patients without lateral ptosis, this fixation step was omitted. Subcutaneous separation and tension reduction were performed, followed by hemostasis. The wound was closed in two layers: 5-0 absorbable interrupted dermal sutures and 6-0 nylon epidermal sutures. A pressure dressing was applied, with sutures removed at day 7. This procedure does not significantly elevate brow height; instead, it reshapes and tightens the upper eyelid area to improve eyelid fold and contour.

### Data collection

Standardized photography was employed for data collection. The same surgeon used the camera (Canon EOS 5D Mark IV camera with an EF 100 mm f/2.8l Macro lens) to take photos at a distance of 1.5 meters from the patient under flash lighting. Consistency in lighting, background, angle (45-degree angles on either side of the patient), and distance was maintained to ensure the comparability of photos. The camera settings were standardized for all photographs: aperture f/8.0, focal length 100 mm and ISO 200. Patients were seated with their heads level, necks in a neutral position, naturally relaxed, and looking straight ahead. The shooting posture was required to be consistent at each stage. Each patient was photographed three times, and photos with high clarity and sufficient resolution showing the details of the eyebrow and eye area were selected. Scale data were completed independently by the patients. Researchers had received unified professional training before the study and used the same instructions to reduce bias. The scales were collected on-site upon completion and 8 surveys were excluded (5 completed in <3 min; 3 showing random patterns) from the recovered scale data.

### Observation indicators

**Figure 1:**
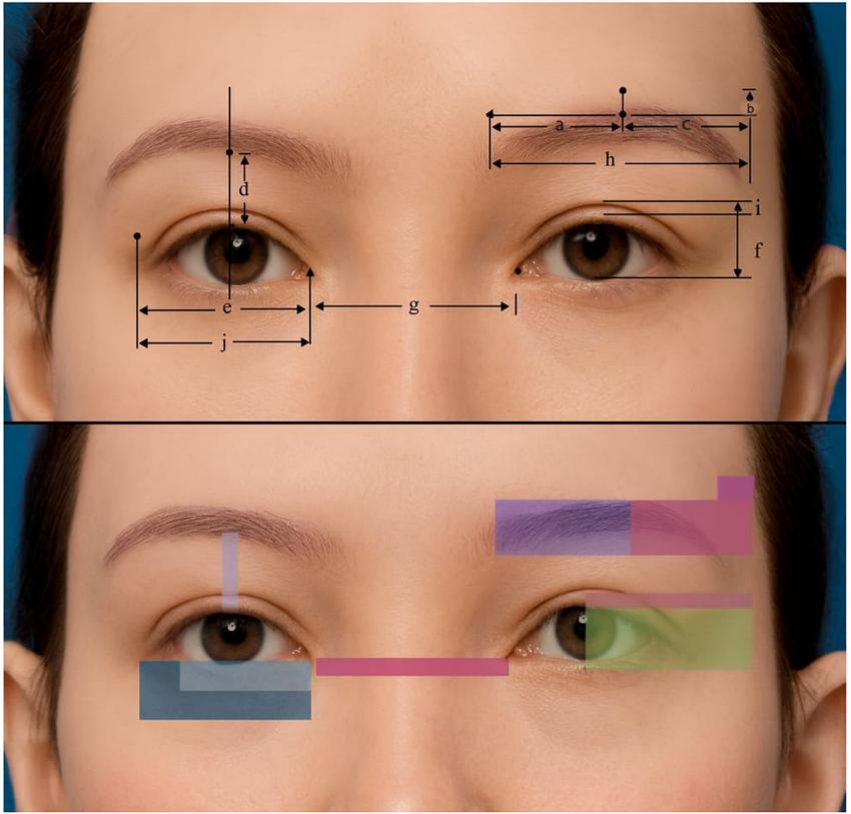
Simulation of standardized measurements and shading overlays used to assess morphological outcomes following sub-brow blepharoplasty. The top panel shows annotated horizontal and vertical dimensions of the eyebrows, with labeled reference lines **(a–j)**. The bottom panel presents color-coded translucent overlays highlighting specific regions of interest. The illustrative base image was generated using an anonymized AI-based image simulation system and does not depict any real patient. Measurement lines, arrows, and shading overlays were created and edited in Microsoft PowerPoint (Office 365). **(a)** horizontal distance from the superior point of the medial brow to the brow peak; **(b)** Vertical distance from the brow peak to a horizontal reference line drawn through the superior border of the medial brow; **(c)** horizontal distance from the brow peak to the lateral brow end; **(d)** vertical distance from the upper eyelid margin at the mid pupil to the inferior edge of the supraorbital ridge; **(e)** horizontal distance between the medial and lateral Commissure; **(f)** vertical palpebral fissure height; **(g)** horizontal distance between the two medial Commissure; **(h)** horizontal distance from the medial brow to the lateral brow end; **(i)** vertical distance from the double-eyelid crease at the pupil to the upper eyelid margin; **(j)** horizontal distance from the lateral end of the double-eyelid crease to the medial Commissure.

Patients were followed up at three post-operative time points i.e., immediately after surgery (T1), one-month post-surgery (T2), and six months post-surgery (T3). All follow-ups were conducted in an outpatient setting and evaluated using standardized photography employing the same camera model, lighting conditions, and patient positioning protocol at each time point. The measurement items are illustrated in Figure 1. Changes in the ratios and dimensions of a/b, a/c, e/f, e/g, d/f, h/e, i/f, d/i, and j/e were measured preoperatively (T0) and at T1, T2, and T3. To ensure consistency, all evaluations were performed by the same surgeon and clinical team.

Psychological status at T0, T1, T2, and T3 was assessed using the Social Appearance Anxiety Scale (SAAS) (10). While the SAAS was originally developed and validated in Western populations, it has also demonstrated robust psychometric validity in Asian contexts. For example, the Indonesian version showed high internal consistency (Cronbach's *α* = 0.932) and good test–retest reliability (ICC = 0.850) (11), while the Korean version reported *α* = 0.95 and test–retest *r* = 0.918 (12). These findings support the reliability of the SAAS for use in our study population. The scale consists of 16 items, scored using a Likert 5-point scale ranging from 1 (not at all) to 5 (extremely), with item 1 being reverse-scored. The individual's appearance anxiety positively correlates with the total score on the scale. In this study, the Cronbach's *α* coefficient of the scale was 0.950.

A subjective satisfaction scale for surgical outcomes was designed according to the Facial Appearance Satisfaction Scale (Table 1) (13). Patients' subjective satisfaction with the surgical outcomes was recorded until the final follow-up (six months post-surgery).

**Table 1:** Subjective satisfaction scale for surgical outcomes by patients.

Subjective satisfaction	Evaluation criteria
Extremely satisfied	Significant improvement in eyebrow ptosis/upper eyelid skin laxity and eyebrow contour, with excellent eyebrow position and natural appearance.
Satisfied	Noticeable improvement in upper eyelid laxity with appropriate eyelid fold and natural appearance.
Neutral	Moderate improvement with acceptable appearance compared to pre-operative state.
Dissatisfied	Insufficient improvement, with unsatisfactory eyelid fold or unnatural appearance.

This scale is adapted from the facial appearance satisfaction scale. Higher levels indicate greater patient-reported benefit.

### Statistical methods

For normally distributed data, results were presented as mean ± standard deviation, while skewed data used the median and interquartile range. The Wilcoxon signed-rank test compared non-normally distributed groups. Repeated measures ANOVA was used for multiple normally distributed groups, and the Kruskal–Wallis test for non-normal data. Statistical significance was set at *α* = 0.05, with *p* < 0.05 considered significant.

## Results

**Figure 2:**
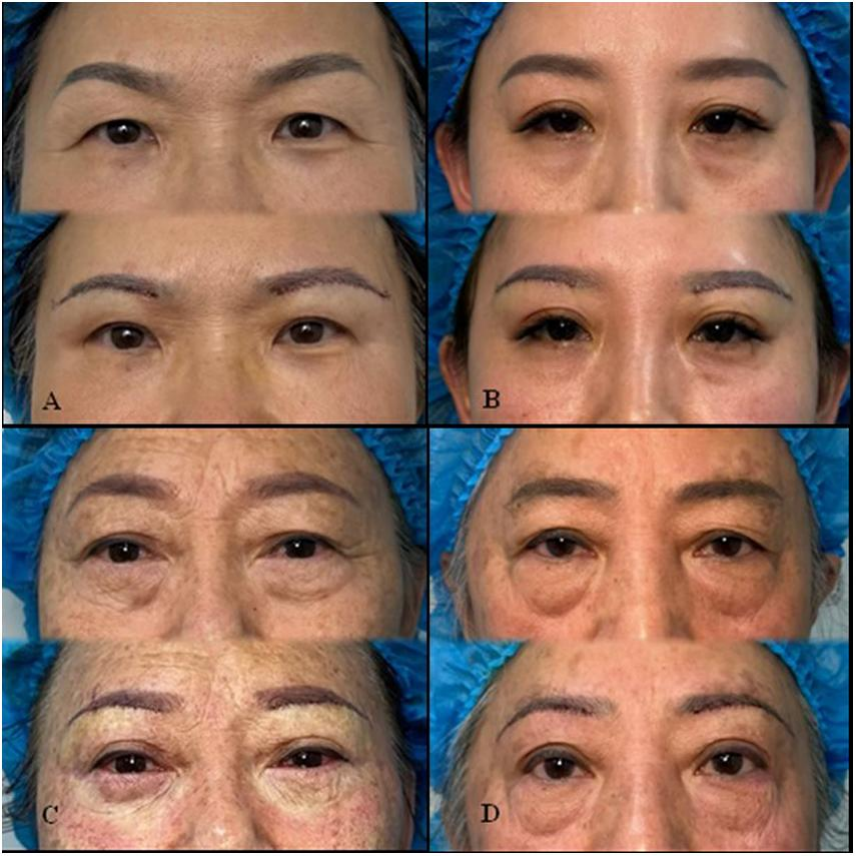
Clinical photographs showing representative morphological outcomes following sub-brow blepharoplasty. Each panel **(A–D)** presents a different patient, with preoperative images on the top and 6-month postoperative images on the bottom. Postoperative results demonstrate improved upper eyelid contour, reduced dermatochalasis, and enhanced brow positioning.

In total, 64 patients were included; 49 completed all follow-ups up to six months (T3), while 15 had follow-ups between 2 and 7 months (mean: 7.5 months). The complete follow-up and clinical data were obtained from 49 patients (98 eyes), resulting in a follow-up rate of 76.56%. The mean ± standard deviation was reported for each measurement, with statistical comparisons assessed using *F*-values and *P*-values. Significant differences (*p* < 0.05) indicated notable changes over time, particularly in variables such as relationship between palpebral fissure height and eyebrow distance (*e/f*), ratio of palpebral fissure height to intercanthal distance (*e/g*), relative brow height compared to eyebrow distance (*d/f*), and brow height to lower lid-to-brow height (*d/i*), suggesting meaningful postoperative effects. While “e” and “g” were expected to remain relatively constant, the observed changes in ratios involving these parameters (e.g., e/f) may reflect indirect effects of soft tissue repositioning rather than true displacement. Notably, the increase in d/f suggests a degree of lid heightening, even in the absence of a formal ptosis correction. Non-significant results, such as proportion of medial to lateral brow length (*a/c*) and horizontal brow span to vertical eye opening (*h/e*), indicated minimal variation across time points (Table 2). Figure 2 presents a comparison of preoperative and postoperative outcomes.

**Table 2:** Changes in eyebrow height at Various measurement points before and after surgery $\left(\bar{x}\pm s\right)$.

Measurement items	T0	T1	T2	T3	*F*-value	*P*-value
a/b	4.26 ± 0.88	4.05 ± 0.67	3.86 ± 0.40	3.75 ± 0.47	5.217	0.007
a/c	2.21 ± 0.48	2.30 ± 0.50	2.49 ± 0.71	2.48 ± 0.61	2.075	0.106
e/f	2.79 ± 0.24	2.70 ± 0.29	2.41 ± 0.20	2.42 ± 0.16	29.935	<0.001
e/g	0.79 ± 0.06	0.70 ± 0.06	0.82 ± 0.04	0.83 ± 0.07	65.517	<0.001
d/f	1.31 ± 0.21	1.32 ± 0.24	0.87 ± 0.21	0.85 ± 0.22	75.496	<0.001
h/e	1.65 ± 0.15	1.63 ± 0.17	1.63 ± 0.16	1.63 ± 0.15	0.956	0.416
i/f	0.28 ± 0.08	0.27 ± 0.06	0.26 ± 0.06	0.26 ± 0.07	3.017	0.032
d/i	4.79 ± 0.86	3.96 ± 0.88	3.87 ± 0.84	3.81 ± 0.82	14.234	<0.001
j/e	1.20 ± 0.05	1.19 ± 0.08	1.18 ± 0.07	1.19 ± 0.09	2.165	0.095

- a/b: Proportion of medial brow to total brow length.

- a/c: Proportion of medial to lateral brow length.

- e/f: Relationship between palpebral fissure height and eyebrow distance.

- e/g: Ratio of palpebral fissure height to intercanthal distance.

- d/f: Relative brow height compared to eyebrow distance.

- h/e: Horizontal brow span to vertical eye opening.

- i/f: Lower lid to brow height relative to eyebrow distance.

- d/i: Brow height to lower lid-to-brow height.

- j/e: Eye width to eye opening height.

- *F*-value/Inter-group = test statistic for between-timepoint comparisons.

Postoperative scores on the Social Appearance Anxiety Scale (SAAS) demonstrated a consistent and significant decline over time, with mean scores decreasing from 56.26 ± 5.14 at baseline to 12.11 ± 3.41 at the final follow-up (Table 3). This downward trend was statistically significant across all time points (*P* < 0.001), indicating a marked reduction in appearance-related anxiety following sub-brow blepharoplasty. All the patients who participated in the study successfully underwent surgery, and no postoperative complications or related adverse events were reported till the last follow-up. According to the subjective satisfaction scale for surgical outcomes, 35 patients (71.43%) expressed extreme satisfaction, 11 patients (22.45%) expressed satisfaction, and 3 patients (6.12%) expressed a neutral attitude. The overall satisfaction rate for the surgery was 93.88%. Pearson correlation analysis demonstrated that changes in the d/I (brow height to lower lid-to-brow height) ratio (*r* = 0.45, *p* < 0.001) and e/f (relationship between palpebral fissure height and eyebrow distance) ratio (*r* = 0.39, *p* < 0.001) were most strongly associated with higher subjective satisfaction.

**Table 3:** Comparison of social appearance anxiety among patients at various measurement points before and after surgery $\left(\bar{x}\pm s\right)$.

Measurement items	T0	T1	T2	T3
Social appearance anxiety	56.26 ± 5.14	39.27 ± 4.89	25.72 ± 4.33	12.11 ± 3.41
*F*-value	753.805			
*P*-value	<0.001			

T0 = pre-operative; T1 = immediately post-op; T2 = 1 month; T3 = 6 months.

## Discussion

The skin of the upper eyelid, which is the thinnest due to a lack of subcutaneous fat and constant micro-movements, is among the first regions to exhibit skin laxity and periorbital aging (14, 15). The clinical consensus is that upper eyelid skin laxity primarily results from elastin and collagen loss in the upper eyelid skin, along with weakened orbicularis oculi muscle function (16, 17). Additionally, anatomical studies across different ethnicities have revealed that Asian populations, characterized by more abundant periorbital adipose tissue and thicker upper eyelid areas, are more prone to developing upper eyelid skin laxity (18). The main clinical manifestations of upper eyelid skin laxity include loss of skin elasticity and gloss, along with alterations in the shape of the eyes, which not only impact patients' facial aesthetics but also restrict their visual fields (19). Removing skin and subcutaneous tissue through incisions below or at the eyebrow is a common procedure for periorbital rejuvenation (20). With the growing emphasis on eyebrow-eye integration, surgeons must consider the morphology, position, and proportions of the eyebrow and eye areas, as eyebrow position and shape are key indicators of eye rejuvenation surgery effectiveness (21). In this study, sixty-four patients undergoing sub-brow blepharoplasty were evaluated preoperatively (T0) and at follow-ups (T1, T2, T3) using standardized photography and measurement of eyebrow and eyelid parameters. Psychological status and surgical satisfaction were also assessed over time. All patients underwent surgery with incisions below the eyebrow. Although this study included only female patients, the technique may also be applicable to male individuals. However, careful preoperative planning is necessary to accommodate gender-specific aesthetic preferences, such as lower eyebrow position and thicker brow hairs in men.

The follow-up rate of patients was 76.56%. The study results showed statistically significant changes over time in the measurement indices (*P* < 0.05) and intergroup effects at each measurement point before and after surgery (*P* < 0.05). According to another study, supra-eyebrow incisions enhance eyebrow lifting, they offer limited improvement in upper eyelid laxity and leave a more visible scar (22). According to previous studies, sub-brow incisions correct eyebrow ptosis by anchoring the fat pad to the periosteum, addressing adhesion abnormalities and weak temporal muscle support (23, 24).

In East Asian populations, the double eyelid crease, a supratarsal fold that contributes to a more defined upper eyelid, is often less pronounced or absent due to unique anatomical characteristics such as lower attachment of the levator aponeurosis, increased preaponeurotic fat, and a thicker orbicularis oculi muscle (25). Cosmetic eyelid procedures in this demographic frequently aim to preserve or enhance the natural crease without causing asymmetry or an unnatural appearance. One of the advantages of sub-brow blepharoplasty in this context is its ability to improve upper eyelid laxity while preserving the native double eyelid fold. By removing redundant skin and soft tissue superior to the crease, without disrupting the tarsal plate or levator complex, this technique helps to maintain or even accentuate the natural contour of the eyelid (20, 26). This preservation is especially significant in East Asian patients, who often place cultural and aesthetic importance on the double eyelid feature.

In the current study, sub-brow blepharoplasty involves suturing and fixing the displaced subbrow fat pad to the supraorbital periosteum. Based on six months of postoperatively follow-up observation data, it can be seen that sub-brow blepharoplasty surgery can effectively improve periorbital aging and laxity. Although the eyebrow height may be slightly affected showing a mild decrease, the overall decline is relatively low, and this degree of change does not affect patients' subjective satisfaction with the postoperative results. Previous studies indicate that combined correction surgeries for upper eyelid skin laxity may result in uneven double eyelid shapes or changes in eyebrow morphology. Additionally, excessive suture tension in sub-brow incisions can lead to secondary eyebrow ptosis (27, 28). After upper eyelid ptosis correction, the compensatory contraction of the frontal muscle weakens, potentially leading to a decrease in eyebrow lifting force and affecting eyebrow height. A single surgical approach may be insufficient, especially for patients with concurrent eyebrow ptosis or naturally low eyebrows, requiring careful consideration of surgical effects on eyebrow height.

While this study focused on patients of East Asian heritage, the sub-brow blepharoplasty technique may also be applicable to non-East Asian populations, particularly those presenting with lateral upper eyelid hooding. In many non-Asian individuals, especially those with a higher supratarsal crease, deeper-set eyes, or less periorbital fat, the sub-brow approach may carry a greater risk of visible scarring and less favorable blending with the brow's natural contour. Additionally, the aesthetic expectations in Western populations may differ, with a greater emphasis on hidden incisions and upper eyelid contouring via the skin crease approach. Therefore, patient selection and ethnic-specific anatomical variation remain critical in determining the appropriateness of the sub-brow technique.

## Conclusion

In conclusion, patients before undergoing sub-brow blepharoplasty surgery often suffer from social appearance anxiety. The surgery can improve the aging state around the eyes, elevate the position of the eyebrows, and positively influence ocular and facial aesthetic morphology. Meanwhile, the results of this study confirm the high safety of sub-brow blepharoplasty surgery, with patients expressing high subjective satisfaction with the surgical outcomes.

## Data Availability

The raw data supporting the conclusions of this article will be made available by the authors, without undue reservation.
