# Supplementary material for: Evaluation of morphological and psychological outcomes after sub-brow blepharoplasty
Source: Front Surg. 2025 Sep 23;12:1658806. doi: 10.3389/fsurg.2025.1658806 (PMC12500621; doi:10.3389/fsurg.2025.1658806)
Supplement: Supplementary file 1 [file Supplementaryfile1.pdf]

Social Appearance Anxiety Scale (SAAS)

Please read each of the following statements carefully and indicate how much each one applies to you in social situations. Use the scale below to rate your level of anxiety or discomfort related to your appearance.

Sr. No.	Items	1	2	3	4	5
1	I feel uncomfortable when I think others are evaluating my appearance.					
2	I worry that others talk about the way I look behind my back.					
3	I am concerned people would not like me because of the way I look.					
4	I am afraid people find me unattractive.					
5	I worry that my appearance will make life more difficult for me.					
6	I feel anxious when I think others are judging my looks.					
7	I feel nervous when having my picture taken.					
8	I get anxious when I have to meet new people because of my appearance.					
9	I am concerned people will judge me when they see me.					
10	I worry that others are unimpressed with my appearance.					
11	I get nervous when preparing to go out in public because of the way I look.					
12	I worry about how others perceive my appearance.					
13	I get anxious when people look at me.					
14	I fear others will find flaws in my appearance.					
15	I worry that people stare at me because of my looks.					
16	I feel self-conscious when I know people can see me.					

1 = Not at all, 2 = Slightly, 3 = Moderately, 4 = Very, 5 = Extremely

Total Score: ________
Minimum: 16, Maximum: 80
Higher scores indicate greater social appearance anxiety.
